# Effects of Oral Appliances for Obstructive Sleep Apnoea in Reduced Periodontium: A Finite Element Analysis

**DOI:** 10.1016/j.identj.2024.05.002

**Published:** 2024-06-04

**Authors:** Manila Caragiuli, Mara Candelari, Francesca Zalunardo, Giovanni Bruno, Alberto De Stefani, Agnese Brunzini, Marco Mandolini

**Affiliations:** a Department of Industrial Engineering and Mathematical Sciences, Università Politecnica delle Marche, Ancona, Italy; b Department of Neuroscience, Dental Clinic, Section of Dentistry, University of Padua, Padua, Italy; c Department of Industrial Engineering, University Tor Vergata, Rome, Italy; d Department of Pharmacological Sciences, University of Padua, Padua, Italy

**Keywords:** Obstructive sleep apnoea, Mandibular advancement device, Finite element method, Finite element analysis, Periodontal disease, Periodontal stress

## Abstract

**Background and objective:**

In the literature, no studies correlate the effects of mandibular advancement devices (MADs) with different titration systems to periodontitis. Through a finite element analysis (FEA), this study investigates the effects generated on periodontal ligaments (PDLs) and teeth by four commercial MADs in periodontal health and with 15% bone resorption.

**Methods:**

Four MADs (Somnodent Flex™, Somnodent Avant™, Orthoapnea™, and Herbst™) were digitalised starting from the impressions of a patient's dental arches. A force of 11.18 N, representing an advancement of 9.5 mm, was applied, and a FEA was subsequently performed. After measuring the stresses and displacements on the PDLs and teeth in healthy periodontal conditions, the vertical dimension of the alveolar bone was reduced by 15%, and measurements were repeated.

**Results:**

In terms of PDL stress, Herbst™ is the device which guarantees a more uniform increment in case of the first stage of periodontitis (+7% for mandibular and maxillary PDLs compared to the healthy condition). For Somnodent™ devices, the PDLs stress increment is almost null for mandibular PDLs but much higher than Herbst™ for maxillary PDLs (+17% and +21% for Flex™ and Avant™). Orthoapnea™ determines a PDL stress augmentation between the other devices (+16% and +7%, respectively, for maxillary and mandibular PDLs). Concerning teeth movement, Herbst™ and Orthoapnea™ determine a lower and more uniform displacement than Somnodent devices.

**Conclusions:**

The stress distribution and teeth displacement are strictly related to MAD geometry. Since its minor effects on teeth and PDLs, the Herbst™ could be more appropriate in patients with periodontitis.

## Introduction

Obstructive sleep apnoea (OSA) is a disorder characterised by recurrent episodes of partial or total upper airway obstruction. Physiological muscular hypotonia occurs during sleep, favouring the airways’ collapse and airflow interruption.^1^ The onset of OSA is dependent on anatomical and functional factors. The patients most predisposed to developing this problem are male adults suffering from overweight or obesity, with mandibular retrusion, micrognathia, or maxillofacial alterations.^2^ Approximately 936 million adults worldwide are believed to be suffering from OSA.^3^

The gold standard of disease treatment is continuous positive airway pressure (CPAP). However, long-term compliance appears to be very poor.^4^ A therapeutic alternative is represented by mandibular advancement devices (MADs). These are dental appliances given by two-maxillary acrylic splints that interface with each other in different ways depending on the mechanism of action. The MAD aims to generate a mandibular protrusion that induces a tensioning of the soft tissues in the retropalatal and retrolingual areas. MAD stabilises the airway and decreases collapsibility.^5^ The effectiveness of oral appliances is comparable to that of CPAP in mild and moderate OSA cases. In severe cases, it is lower, but the good compliance of the patients represents the strength of the MAD.^6^ MADs are easy to make but cannot be used in all patients. There are some contraindications to their use in patients suffering from severe joint problems, periodontal disease in the active phase, grade II or III dental mobility, an insufficient number of healthy teeth that can give retention to the device, or poor jaw movement in advance (<6 mm).^7^

Periodontitis is a pathological condition in which inflammation causes periodontal attachment loss. The most recent analyses establish that periodontal disease in Europe is widespread in the moderate form in 33% to 50% of adults, while more severe cases are found in 17% to 35%.^8^ The situation appears similar in the USA. Data show that 42.2% of the population is affected by periodontitis, with a severe degree occurring in 7.8% of the subjects.^9^

Given the extreme spread of periodontal disease and the contraindications of using MAD, the authors decided to focus on this topic.^10^ To overcome the ethical limits that often condition research in the medical field, it was decided to exploit the finite element method (FEM) to conduct this study. There are several finite element analyses (FEAs) performed on temporomandibular joints,^11^^,^^12^ orthodontic movements,^13^^,^^14^ mini-screws,^15^ teeth restoration,^16^, ^17^, ^18^ and oral device treatment for OSA,^19^ but also in the field of periodontics.^20^

Recent studies investigated the effects of splints on teeth for patients with periodontitis. The work of^21^ evaluated the impact of periodontal splints made from different materials on stress distributions in compromised periodontal tissues (up to 75% of bone loss). Twenty-five FEM models were developed to evaluate the effects of dental splits, which differ from MADs. Another research work presenting a FEM model to analyse patients with periodontitis is given in.^22^ The authors investigated the relationship between bone resorption and dental stress by considering three loading configurations. The work does not consider MADs. In adult patients with various levels of bone loss, Bica et al.^23^ assessed the stress and displacement during the application of orthodontic pressures. The displacement values were discovered to have dramatically risen with bone resorption. Moreover, bone loss alters the stress distribution at the apex of the tooth. It lowers the resistance at the tooth's centre.

Geramy et al.^24^ evaluated orthodontic tooth movement in a bone loss situation (up to 4.5 mm). The FEM model accounts only for a tooth (upper central incisor) without reference to MADs. In another study, the same authors^25^ assessed the displacement of the tooth while applying a constant force (1 N amplitude) and simulating various stages of bone loss (from 1 to 8 mm). The findings showed that alveolar bone loss is associated with an increased moment/force (M/F) ratio needed to induce body movement. As bones are lost, the centre of resistance shifts closer to the apex, reducing the distance from the alveolar ridge.

Kumar et al.^26^ examined the initial stresses in teeth, periodontal ligaments (PDLs), and bone when a force was applied for intrusion and tipping on the labial and lingual sides of the tooth. In contrast, different levels of bone height were present. Six 3D finite element models for six bone heights of a maxillary central incisor with PDL were developed for this investigation. Researchers exerted pressure on the teeth in each model. The analysis was performed considering only a single tooth. The effects of force magnitudes on the long-term tipping movements of teeth under various levels of bone loss were demonstrated by Zargham et al.^27^ Based on a theory of bone remodelling, the long-term tooth movements were reproduced over four weeks. The authors used forces of 1 N to cause and measure tooth displacements and 0.25 N to measure tooth rotations. According to,^28^ additional factors influence the stress on teeth and PDLs in periodontitis. The authors presented the consequences of a maxillary central incisor labiolingual inclination. The objective was to determine if labial tooth inclination and alveolar bone loss impact the moment per unit of force (M/F) during controlled tipping and the resulting stresses on PDLs.

Most research articles that give numerical models for assessing the impact of periodontitis staging on PDLs and teeth consider only one tooth at a time. Moreover, there is no research aiming at correlating the effects of MADs (with different titration systems) to periodontitis (bone resorption). The present study performs a comparative FEA to evaluate the effects of MADs on PDLs and teeth by simulating a reduction of dental bone support due to periodontitis. The study accounts for four commercial MADs designed for a specific patient. Effects were evaluated considering two configurations: the healthy and first stage of periodontitis (15% bone resorption).

## Materials and methods

The finite element model was developed through the following preprocessing steps: 3D Model reconstruction, the definition of material properties, boundary condition settings (constraints, loads, and contact behaviour), and mesh generation.

### 3D Model reconstruction

**Fig. 1:**
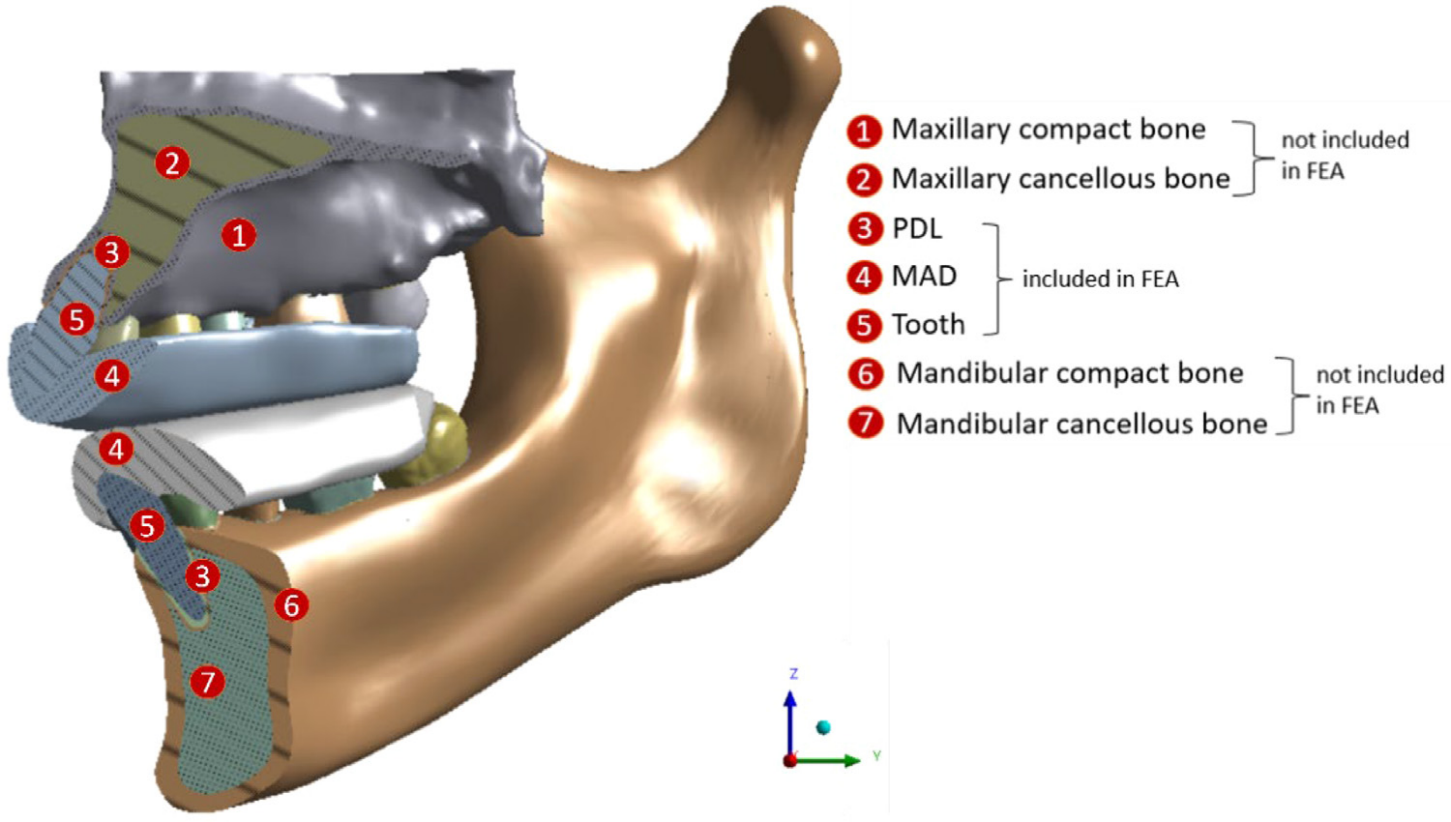
Sagittal section of the reconstructed geometric model.

For the study, the first stage of periodontitis was created according to a modelling procedure starting from an anatomical model belonging to a 29-year-old female patient with health status. The acquired tomography scans (Voxel size 75 micron, FOV 11 × 13 cm NewTom Giano, Cefla) were reconstructed through an image processing software (Mimics v.12.11, Materialise NV) using an appropriate threshold based on the Hounsfield unit of the dense bodies. Since they are not identifiable in computed tomography (CT) scans, soft tissues (e.g. PDLs) were reconstructed through computer-aided design (CAD) software for 3D modelling (Rhinoceros v.5.0 by McNeel & Associates). In particular, the PDL was modelled as a thin layer, 0.3 mm thick, wrapped around the tooth root to fill the space between each tooth and the alveolar socket (Figure 1).^29^

**Fig. 2:**
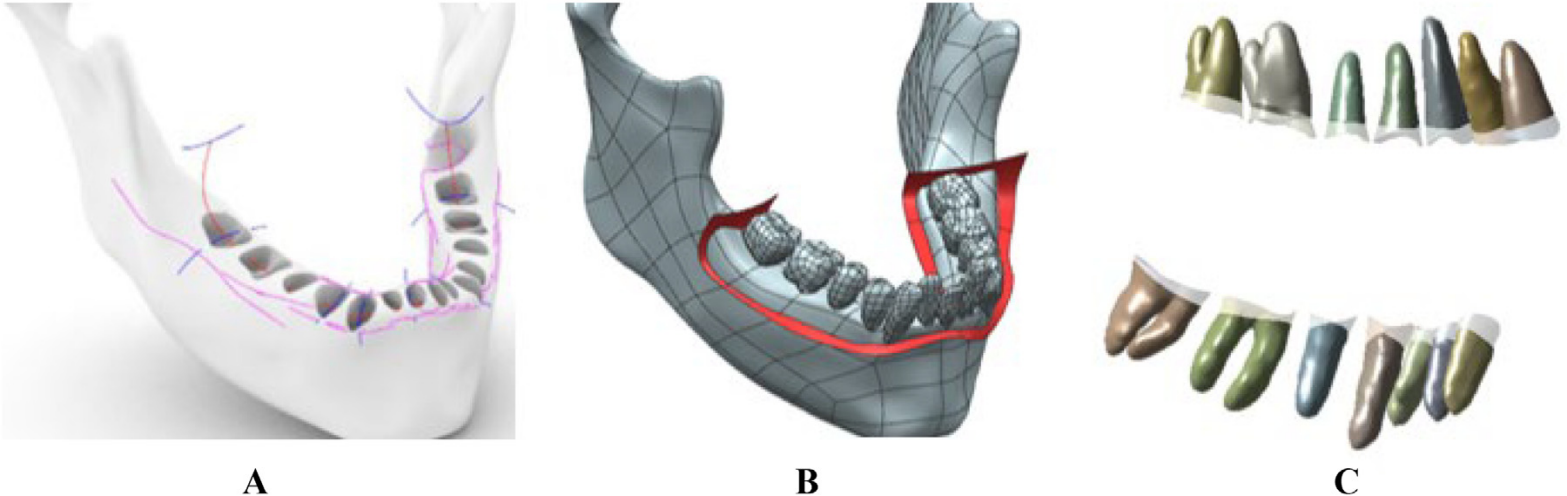
Periodontitis modelling operations. Profile curves (red and blue) set at 15% of the tooth root (A) were interpolated to define the red cutting surface (B) to mimic the effects of the first stage of periodontitis on PDLs (C). In transparency, the healthy model, whereas shaded the pathological one.

The effects of periodontitis were modelled through morphing operations of PDLs and bones, as described in.^29^^,^^30^ In particular, a cutting surface interpolating construction points set at 15% of the tooth root length, measured from the cementoenamel junction to the apex (Figure 2A and B), allowed the removal of an amount of tissue corresponding to the first stage of periodontitis.^31^ The amount of tissue removed is not constant for all the bodies since it depends on the height of each tooth root. Still, the average value is about 15% of the original volume. In Figure 2C, it is possible to appreciate the PDL tissue reduction associated with the pathological condition.

**Fig. 3:**
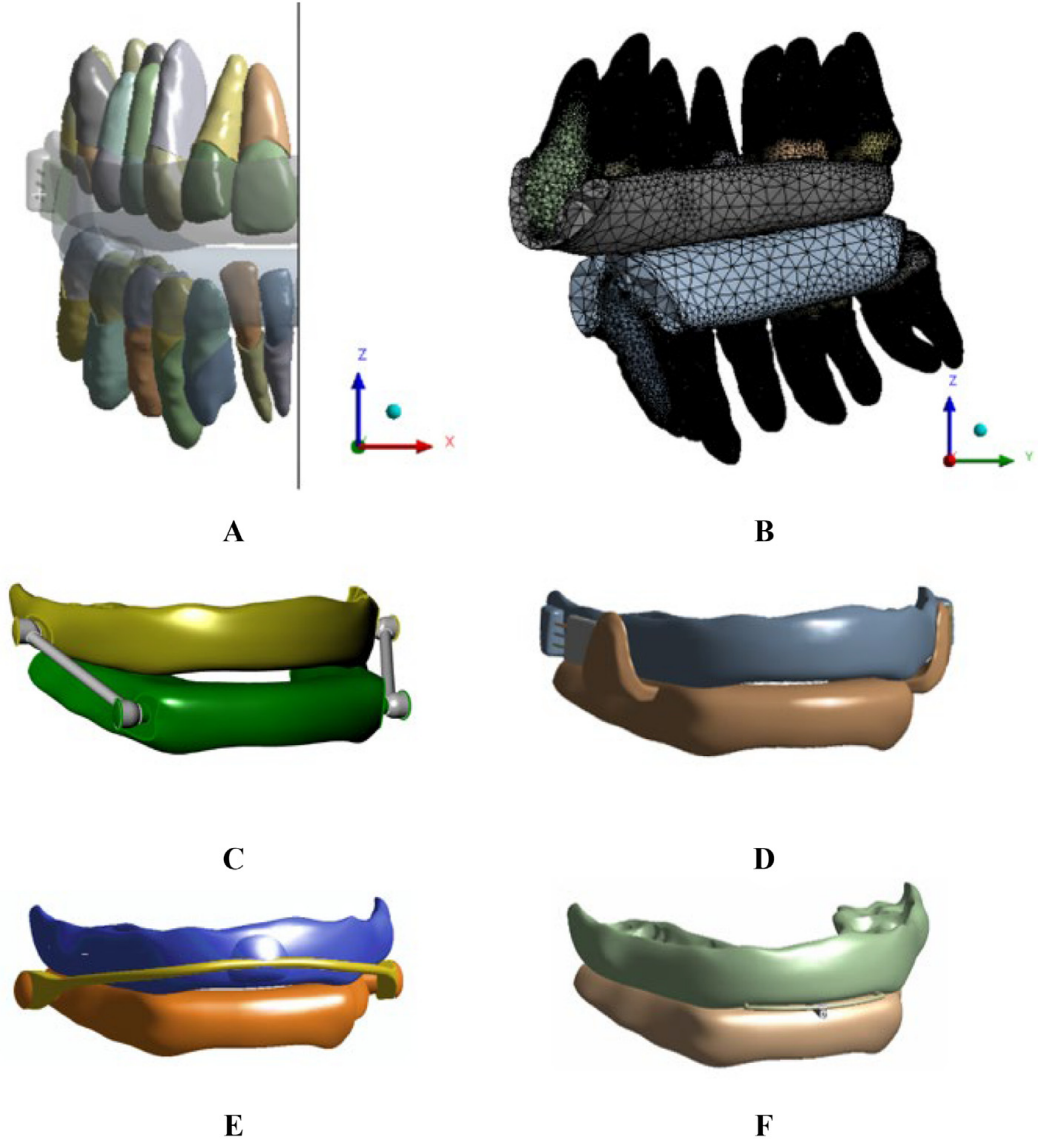
(A) Front view of the 3D finite element model, (B) sagittal view of the mesh details (top). CAD models of four MADs: Herbst™ (C), Somnodent Flex™ (D), Somnodent Avant™ (E), Orthoapnea™ (F) (bottom).

The final model for the FEA included the two dental arches (upper and lower complete teeth), the PDLs, and a MAD (Figure 3A). To reduce the computational effort during the simulation, a symmetric model was assumed by considering the midsagittal plane YZ as the symmetry plane. This allowed to model only half of the model assuming symmetric results on the opposite side and prevented any displacement along the direction normal to the symmetry plane. The maxilla and mandible were neglected due to the higher stiffness compared to PDLs, at least two thousand times stiffer.^32^ Moreover, the authors verified that the integration of the mandibular bone components (cortical and cancellous bone) did not provide any difference in the stress results of the PDLs with respect to the simplified model discarding the bone. Hence, it is reasonable to carry out the study by constraining the outer surface of the PDLs. Thus, the effects of periodontitis can be appreciated on PDLs (Figure 2C). Four MAD models with different coupling mechanisms were designed following a reverse engineering approach (Figure 3). Two telescopic side arms characterise an Herbst™-like MAD (Figure 3C). This MAD has a connection mechanism between the upper posterior teeth and the lower canine area. The Somnodent Flex™ splints are connected by an adjustable interlocking inclined buccal extension pushing against mandibular fins (Figure 3D). Somnodent Avant™ (Figure 3E) has a strap mechanism linking the lower molars to the maxillary frontal group. The shorter the strap, the higher the mandibular advancement. Orthoapnea™ (Figure 3F) is a bimaxillary device with an anterior reverse connecting rod mechanism. MAD CAD models are not commonly available since they are tailored to the individual and handcrafted by practitioners. Thus, an optical laser scanner (Konica Minolta Range 7) was used for digitising their physical prototype to be subsequently modelled for further refinement. Indeed, by performing multiple laser scans of the physical model of each MAD, it was possible to convert the acquired point clouds into a mesh and, finally, a surface. For a reasonable comparison among the devices preventing bias in the 3D CAD model reconstruction, the design procedure of each MAD component was based on a template model including only the splints of one single MAD. The splint morphology was adapted to the patient's teeth through Boolean operations to guarantee a perfect matching between the components. Then, each splint was optimised according to the specific features of each MAD (rods, arches, fins, telescopic arms, etc.). This way, four 3D CAD models of a MAD have been obtained, preserving each MAD's morphology, the fitting to the patient's dental arches, and the alignment to the overall geometry.

The geometric model was finalised for subsequent FEA in Ansys 2022 R1.

### Material properties definition

For the material properties, linear elastic (based on Hooke's law) and isotropic (the same elastic properties in all directions) materials were assumed with specific Young's moduli and Poisson's ratio (Table). Although the four MAD models differ in design and material, a generic hard acrylic material was assumed to conduct a comparative analysis for all the devices. Concerning PDLs, the nonlinear nature was represented by a hyperelastic model behaviour described through a strain energy density function reported in the following Eq. 1:

(1) $$W=\sum_{i=1}^{N}\frac{2\mu_{i}}{\alpha_{i}^{2}}\left({\bar{\lambda }}_{1}^{\alpha_{i}}+{\bar{\lambda }}_{2}^{\alpha_{i}}+{\bar{\lambda }}_{3}^{\alpha_{i}}-3\right)+\sum_{i=1}^{N}\frac{1}{D_{i}}{\left(J-1\right)}^{2i}$$

**Table:** Material properties used in the finite element model.

Material	Model	Coefficient values
Acrylic MAD	Linear isotropic elastic	*E* = 8,300 *ʋ* = 0.28^34^
Tooth	Linear isotropic elastic	*E* = 18,300 *ʋ* = 0.31^35^^,^^36^
PDLs	Hyperelastic-Ogden	*α*_1_ = −3.4761 *α*_2_ = 18.679 *µ*_1_ = −0.034004 *µ*_2_ = 0.00088691 *d*_1_ = 0 *d_2_* = 0

MAD, mandibular advancement device; PDLs, periodontal ligaments; *E*, Young's modulus (MPa); *ʋ,* Poisson's ratio.

*W* is the strain energy function, *λ*_1_*, λ*_2_*, λ*_3_ the principle stretches of the left Cauchy–Green tensor, and *J* is the determinant of the elastic deformation gradient. The parameter *N* is the order of the polynomial. The constant *µ_i_* is related to the initial (i.e. around the undeformed configuration) shear modulus of the material, and the coefficients *D_i_* to the initial bulk modulus of the material. Finally, the parameters *α_i_* affect the nonlinearity of the stress-strain relationship and make the model capable of fitting experimental stress-strain curves that show a strong nonlinearity. By fitting the uniaxial experimental data based on the work of Natali et al.,^33^ it was possible to derive the parameters listed in Table. In particular, the second-order Ogden model best fitted the data.

### Boundary conditions settings

Concerning the boundary conditions to be applied to the finite element model, specific constraints and contact pairs have been defined to prevent rigid body motion for the static structural analysis. The maxillary and mandibular PDLs were constrained in the space through fixed support to mimic the effect of bone, thus avoiding a rigid motion without deformation. Linear contacts have been defined between the bodies.

A *bonded* contact was defined between PDLs and teeth and teeth and MAD to prevent slippage or separation between the surfaces. Indeed, the two interfaces act as glued bodies to ensure a proper anchorage. PDLs provide adequate support to the tooth to withstand the pressure from grinding or clenching. On the other hand, MAD splints fit over the teeth due to the interproximal space guaranteeing proper retention on the dental arches and keeping the mandible advanced. Despite this contact not being physically representative, the retention force will be greater than the applied load, preventing any dislodgement of the teeth. A *no separation* contact was used between the two splints of the MAD since they are coupled in a configuration that allows mutual sliding without separation. This type of contact differs from the previous one since the two bodies can slide over each other. However, despite the contact surfaces being flat, a static structural simulation was performed by applying an overall protrusion force corresponding to an advanced positioned MAD, as later described.

**Fig. 4:**
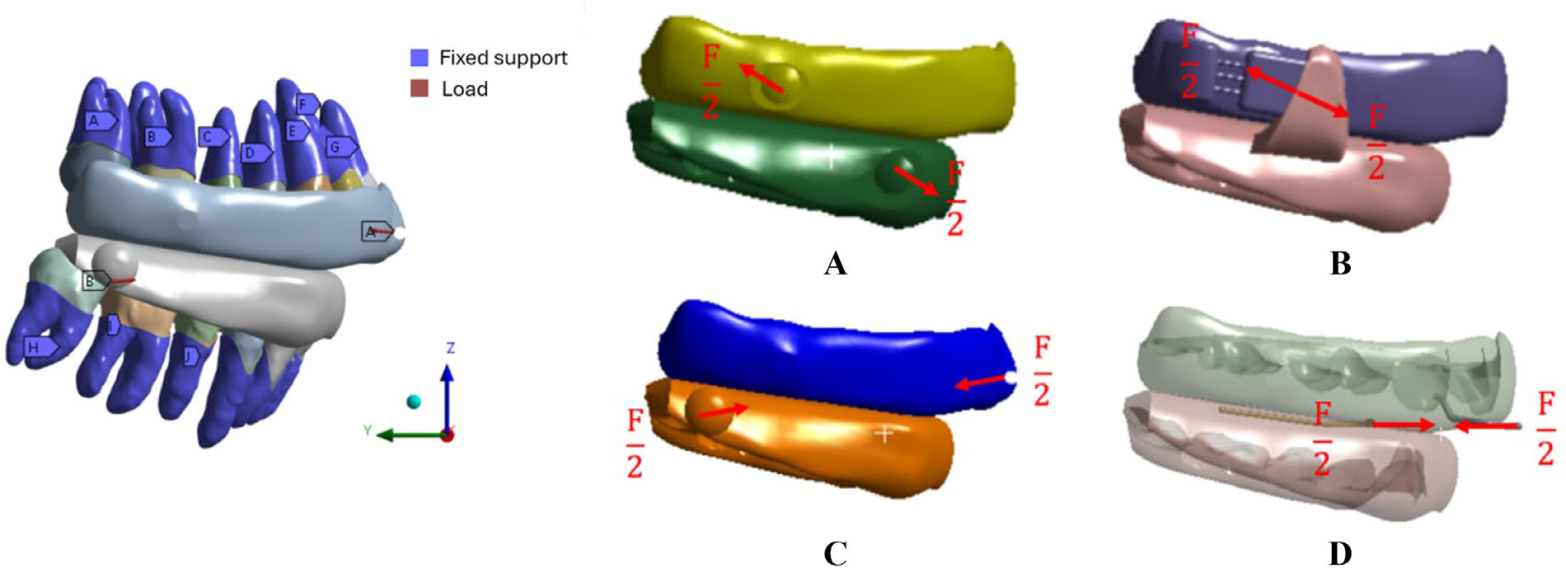
Boundary conditions representation (left). Point of application of the forces represented by red arrows (right): Herbst™ (A), Somnodent Flex™ (B), Somnodent Avant™ (C), Orthoapnea™ (D).

Although the mandibular position depends on the interaction with the oral appliances and the temporomandibular joint, given the complexity of the biomechanical system, the authors assumed a force boundary condition to define the simulation model. In particular, to carry out comparative static structural analyses, the MADs were set to allow a mandibular advancement of 9.5 mm (measured according to the procedure developed by Bruno et al.,^37^) which is in the range of OSA's treatment.^38^ Due to the simulation's static nature, equal and opposite forces of 5.59 N were applied at the connection side between the splints of each MAD. The direction of the contact element between the splints gave the direction of the force vector (Figure 4). The force load was identified based on the force values measured by a pressure transducer during a progressive mandibular advancement^39^ and assumed consistent for each device for a reasonable comparison among the models.

### Mesh generation

Eight simulation models (healthy and pathological models for each MAD) were defined by sharing the same settings to compare the results reasonably. The discretisation phase assumed a tetrahedral mesh with linear elements suitable for complex geometries. A mean mesh size of 1 mm allowed to discretise the 3D bodies, and a smaller size of 0.2 mm with curvature-based refinement entitled to obtain a dense and tight mesh in the thickness of the PDLs despite their thinness. In particular, a mesh convergence analysis was performed in a previous study^40^ to determine the best size for PDLs’ elements as a trade-off between mesh quality and computational resources. It was possible to decide on the optimum element size by evaluating changes in the equivalent von Mises stress while decreasing the element size. This way, getting an accurate and high-quality mesh was possible without jeopardising the workstation's performance regarding computational effort (Figure 3B).

## Results

A comparative analysis has been carried out through FE simulations performed on four MADs’ healthy and periodontal models. The results mainly focus on the findings related to the MAD design and the effects of the first stage of the pathology compared to a healthy condition.

**Fig. 5:**
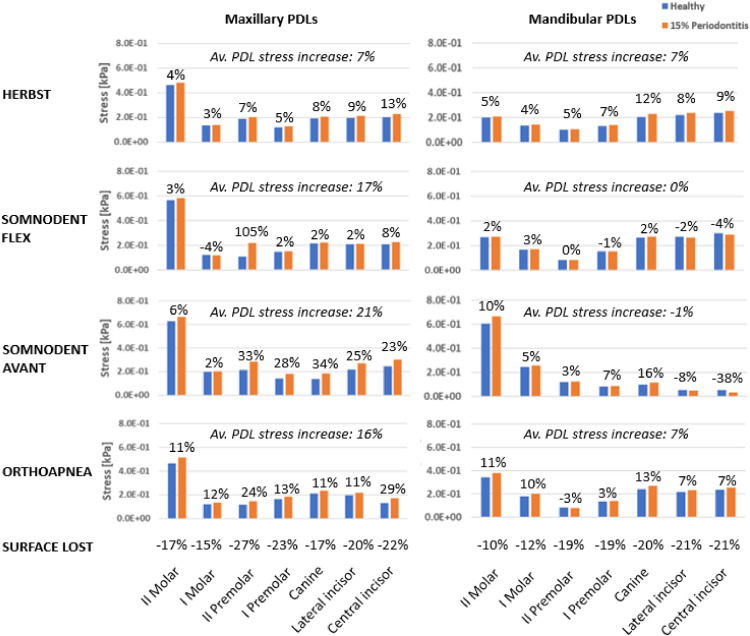
Average stress (kPa) on PDLs of maxillary (left) and mandibular (right) PDLs showing the difference between healthy (blue) and pathological (orange) models. ‘Av. PDL stress increase’ refers to the increase in stress between healthy and pathological models averaged on the PDLs of the maxillary or mandibular arch. Each row refers to a specific MAD. The last row reports the percentage of PDL tissue reduction due to the first stage of periodontitis.

The effects of periodontitis have been investigated in terms of stress on PDLs and teeth and dental displacements. In particular, the average value of von Mises equivalent stress^41^^,^^42^ was assumed as the primary indicator to measure the stress on PDLs, as already done in previous studies^43^ (Figure 5). von Mises considers multiaxial stress acting on a determined point; this allows for a more accurate prediction of structure behaviour under load.

Moreover, it was demonstrated that a high value of stress on PDLs can lead to alveolar bone tissue remodelling, causing malocclusion and teeth displacement. For this reason, previous studies have tried to identify the onset stress value that triggers bone remodelling. However, it has not been identified yet. Penedo et al.^44^ have set the target at 2.60 kPa, while Hohmann et al.^45^ have referred to hydrostatic pressure, demonstrating that over the 4.70 kPa, the stress is correlated with root resorption for teeth. Further investigations are needed in this way.

From Figure 5, it is possible to note the amount of PDL tissue lost due to the first stage of periodontitis (15%).

**Fig. 6:**
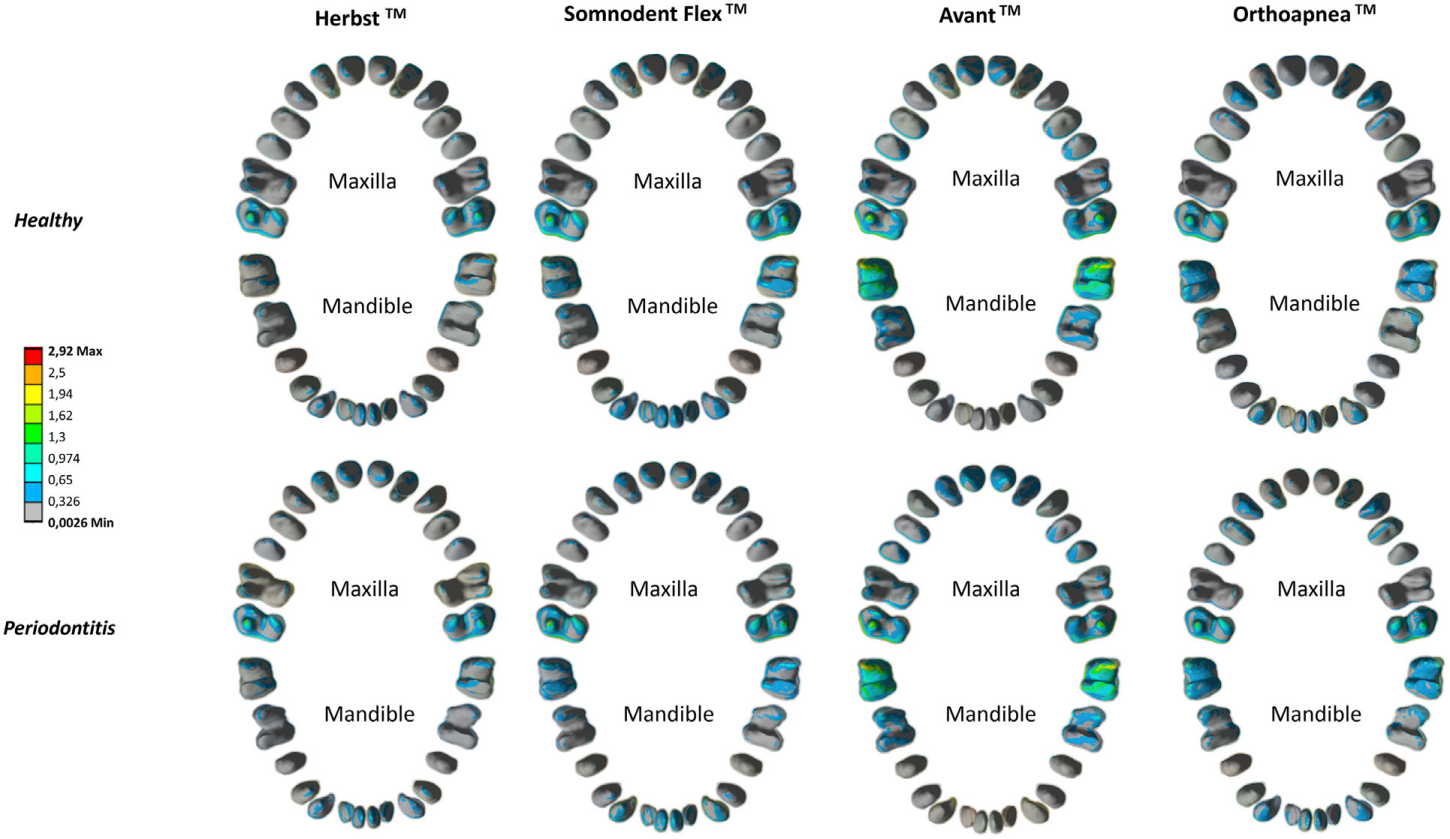
Colourimetric map of the stress distribution (kPa) of the inner surface of PDLs across the four MADs healthy (top) and pathological (bottom) models.

Figure 6 reports the von Mises stress distribution of the inner surface of the periodontal ligaments.

**Fig. 7:**
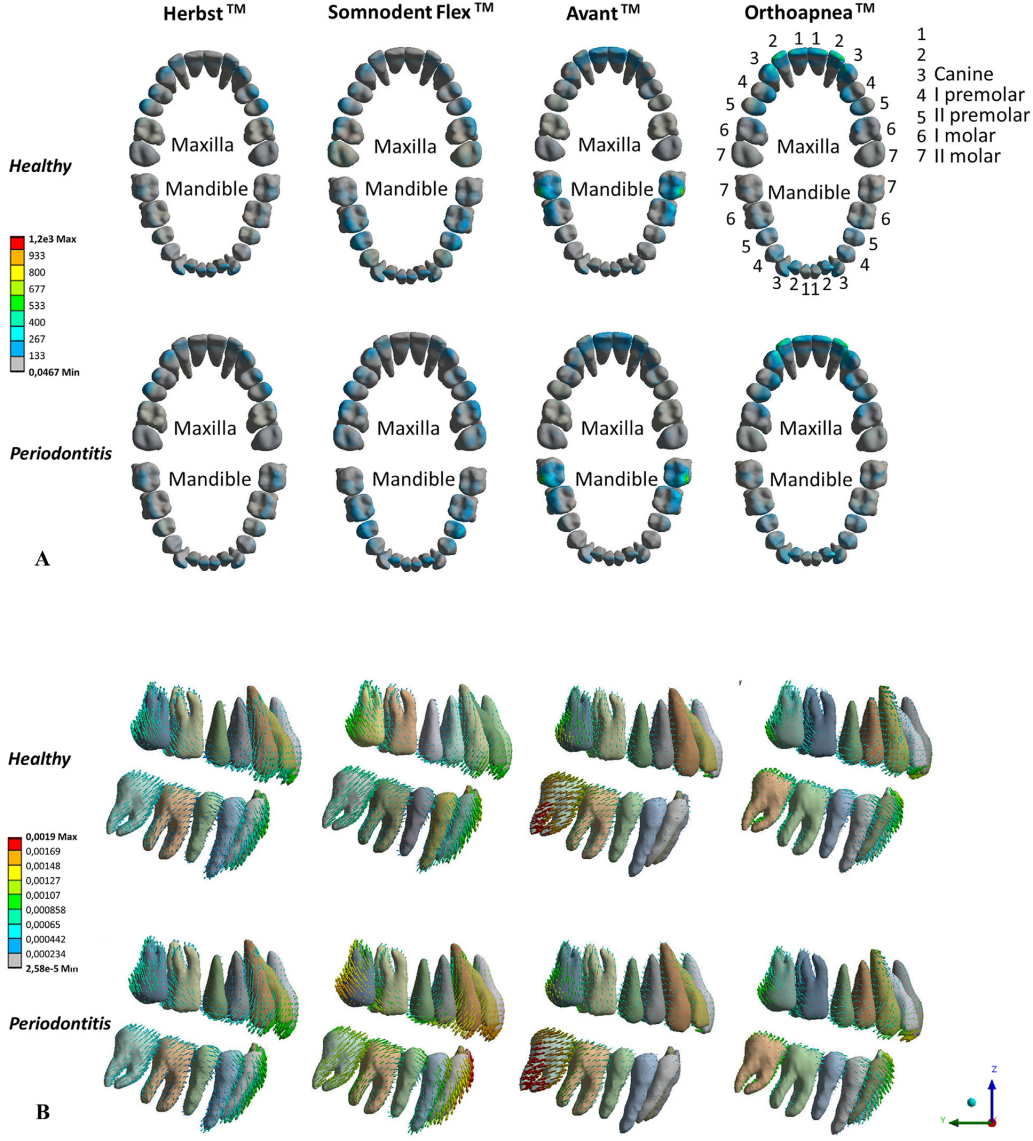
(A) Occlusal view of maximum von Mises stress (kPa) distribution on teeth of four MAD's healthy (top) and pathological (bottom) models, (B) lateral view representing dental displacement (mm) among four MAD designs of healthy (top) and pathological (bottom) models.

Concerning the teeth, Figure 7A displays the occlusal view of the maximum von Mises stress distribution on both dental arches among the eight FE models. The authors decided to pay attention to dental stress distribution because it causes orthodontic teeth movements, which are strictly related to injuries to the periodontium. In health conditions, the alveolar bone adapts itself to mechanical strains; instead, an increasing load on teeth, and thus on PDLs, is associated with tension and compression at levels of nerve ending and blood vessels, hereby inhibiting the preosteoclasts recruitment, indispensable for bone resorption during teeth movements. When the load on teeth is prolonged, it causes acute inflammation that becomes chronic.^46^^,^^47^

Figure 7B represents the teeth displacement through a vector-based view. The arrows point to the direction of the movement, and lengths are proportional to the magnitude.

## Discussion

This work came from the need to enrich the literature about the MAD effects in the case of periodontitis by analysing, through FEM, the stress and displacement exerted by four commercial MADs on PDL and teeth. Most studies investigate a single tooth subjected to a specific load by comparing the effects of different bone heights. Instead, the present study aims to simulate a complete model where exerted forces depend on MAD geometry.

The outcomes of this work have demonstrated that the stress distribution and teeth displacement are strictly related to the MAD geometry. The connection sites between the splints are associated with more significant von Mises stress and tooth displacement values. This result is visible in Figure 7A, where MADs with frontal connections have von Mises stress peaks, mainly in the anterior teeth. Somnodent Avant™ focuses its stress values at the vestibular level of the lower molars and vestibular and palatal surfaces of the superior incisors. At the same time, the rest of the elements are solicited modestly. Moreover, the stress peaks are associated with teeth displacements. Indeed, as reported in Figure 7B, the highest displacement value (0.0019 mm) is associated with the lower molars. Parallelly, the anterior reverse connecting rod mechanism of Orthoapnea™ results in the stress concentration on the anterior elements and premolars teeth and teeth displacement (maximum value of about 0.0012 mm, as in Figure 7B). These results are supported by the study of Uniken Venema et al.,^48^ who investigated the changes in dental occlusion determined by two types of MAD. The MAD characterised by an anterior protrusion mechanism produces more pronounced occlusal changes (overjet and anterior/posterior mandibular movement) than a bilateral thrust MAD.

In contrast, in Somnodent Flex™, the MAD with a lateral mechanism, the stresses induced are of lower magnitude than the previous two systems. Besides, the stress appears more evenly distributed, concentrating on the maxillary molar and mandibular premolar area, whilst the displacement primarily affects the maxillary molars and the mandibular area of the canine and the lateral incisor (0.0012). Lastly, maxillary premolars and the mandibular anterior region are the most affected in the Herbst™, where the splints connect the upper posterior teeth with the lower anterior teeth (Figure 7A). However, in correspondence with this solicited area, Herbst™ have demonstrated the lowest value of displacement, about 0.0011 mm (Figure 7B). Therefore, the stress distribution and teeth displacement are strictly related to the type of connection mechanism between MAD splits. These results, related to healthy models, agree with those already obtained in 2021.^49^

Furthermore, it is possible to note a slight increase in stress and displacement in the case of periodontitis, which can be better appreciated by analysing the PDLs (Figure 5). From the outcomes, it is notable that periodontitis is associated with more significant stress on the maxillary PDLs, especially in the Somnodent Avant™, in the Orthoapnea™ and in the Somnodent Flex™, where the most significant growth in mean stress is shown with an increase of 21%, 16%, and 17%, respectively (Figure 5). The lowest stress value in the maxilla is exerted by the Herbst™ (7%). A different situation is represented in the mandible, where the highest value of stress is generated by Herbst™ and Orthoapnea™ (7%). Instead, others have no significant changes (0%, −1%). The Somnodent Avant™ causes more emphasised stress, localised in correspondence with upper and lower molars with values above 6 × 10^−1^ kPa (Figure 5). In agreement with the results previously reported for teeth stress, the most solicited area of PDLs is still in correspondence with splints connection, except for upper molars.

From a qualitative perspective, the trend of stress distribution of the inner surface of PDLs reproduces that one of teeth and is comparable between the healthy and pathological models (Figure 6). Avant™ and Somnodent Flex™ anterior maxillary PDLs and Orthoapnea™ PDLs report the greatest evidence of stress increase in the pathological model. The pathological model generally shows a higher stress value probably caused by the PDL surface loss. This result is more evident in the maxilla than the mandible since maxillary PDL tissue loss is more significant (mean value −20% vs −17%).

The research findings are supported by Bica et al.,^23^ who analysed the stress and displacement of an upper incisor tooth under different forces in case of a progressive bone reduction of 0, 2, and 4 mm. In addition, Kumar et al.^26^ investigated the stress when applying intrusive and tipping forces on a maxillary central incisor in the presence of different bone heights. The results of these authors confirm an increase in stress and displacement in the pathological model. Geramy^24^ demonstrated the stress increase of a maxillary incisor with a progressive reduction in bone height. A more recent work^22^ studied the effects of vertical and oblique loading on different stages of periodontitis, ranging from healthy conditions to severe periodontitis. The results show an increase in the stress of each structure of periodontal tissue with the degree of alveolar bone resorption.

Observing stress distribution patterns and dental displacements resulting from different MADs carries significant clinical implications for both teeth and PDLs. It has been observed that MADs with frontal connections tend to exert more considerable stress on anterior teeth, potentially leading to slight displacements or changes in their position. These alterations can have implications for patients’ dental occlusion and aesthetics. Additionally, MADs like the Somnodent Avant™ that concentrate stress on the vestibular level of lower molars and the vestibular and palatal surfaces of superior incisors may influence the stability of these dental regions, warranting particular attention during follow-up examinations.

Moreover, even though the changes in tooth position are minimal, over time, they could contribute to alterations in occlusion, with potential functional and aesthetic implications for patients.

MAD therapy can impact PDL tissues, especially with devices causing stress concentration on specific dental elements, highlighted by maximum stress distribution on specific dental crowns. Increased stress on PDLs may worsen periodontal conditions, especially in patients with preexisting periodontal disease. Rapid and uncontrolled tooth movement of one or more teeth may occur in this condition. Pathological alterations of the periodontium cannot be excluded even in healthy patients; therefore, the state of health requires continuous long-term follow-up.

Although the results of this study are coherent with the actual effects of MADs and periodontitis, the simulated model included a few limitations. The first approximation is related to the MAD material. Due to a lack of specific material properties of MADs, the same material was assumed for all cases. A further limitation refers to the simulation process. Mandibular advancement is an incremental process where clinicians gradually increase the mandibular protrusion. In the proposed simulation model, the force applied on the MAD refers to the advancement of 9.5 mm enforced in a single step and a static configuration. Another consideration is the symmetry of the model. Even though the values of the left side may differ slightly based on the physiological differences, a good approximation has been achieved, significantly reducing the computational effort of the simulations. Although the bonded condition assumed between the teeth and the MAD represents a limitation that is not representative of reality, it is reasonable since the interproximal space between the teeth (that characterises the shape of the device) makes the retention force great enough to withstand the protrusion force. The benefit is a faster convergence of the simulation. Moreover, any tensile tractions that could develop to the tooth should be assessed in relative terms, not absolute ones, because of a consistent setup for all the FE models.

Concerning periodontitis, this study modelled the bone loss in a homogenous way, neglecting the local differences between teeth. Moreover, the loss of clinical attachment or the probes is not reproducible because the soft tissues are absent in the model.

In conclusion, MAD with a lateral mechanism is preferable to MAD with an anterior one because the stress is more homogeneously distributed. Due to its lower values of displacement and stress on both teeth and PDLs, Herbst™ can be more appropriate in periodontitis.

In contrast, Somnodent Flex™ and Orthoapnea™ are strongly discouraged in periodontitis because they could worsen pathological conditions.

## Conclusion

The paper investigated and compared the effects of commercial MADs for a patient with the first stage of periodontitis (15% bone resorption). The FEA allowed the evaluation of stress and displacements on teeth and PDLs.

Regarding PDL stress, Herbst™ is the device that guarantees a more uniform increment in case of the first stage of periodontitis (+7% for mandibular and maxillary PDLs compared to the healthy condition). For Somnodent™ devices, the PDLs stress increment is almost null for mandibular PDLs but much higher than Herbst™ for maxillary PDLs (+17% and +21% for Flex™ and Avant™). Orthoapnea™ determines a PDL stress augmentation between the other devices (+16% and +7%, respectively, for maxillary and mandibular PDLs). Concerning teeth movement, Herbst™ and Orthoapnea™ determine a lower and more uniform displacement than Somnodent™ devices (around 0.0012 vs 0.0019 mm). Since its lower values of displacement and stress on teeth and PDLs, the Herbst™ could be more appropriate in periodontitis.

The study has significant clinical implications for teeth and PDLs. MADs concentrating stress on specific dental regions may lead to long-term changes in occlusion. PDLs are also impacted, especially in patients with preexisting periodontal disease, necessitating continuous long-term health monitoring.

Future work first aims to widen the dataset of results. Indeed, in the present work, the authors have set the same PDL properties for all cases; however, this assumption represents a limitation because periodontitis could change the PDL features. Thus, further investigations are needed in this way. Also, this study investigated only the first stage of periodontitis. Further development could be devoted to exploring more severe stages of periodontitis (defined in Tonetti et al.^31^) compared to a healthy status for a more comprehensive comparison of MADs.

Furthermore, bone resorption was simulated with a systematic modelling procedure based on uniform bone loss among teeth. In future work, intertooth variability should be considered for bone resorption modelling. CT scans of pathological patients must be analysed to increase the truthfulness of 3D geometries. The simulation model can be improved because the OSA treatment gradually increases the mandible advancement. Thus, the mandible protrusion force will be stepwise applied.

## Conflict of interest

The authors confirm that this work is original and has not been published elsewhere nor is it currently under consideration for publication elsewhere. The authors declare that they have no known competing financial interests or personal relationships that could have appeared to influence the work reported in this article.
